# Prognostic biomarkers and therapeutic targets in oral squamous cell carcinoma: a study based on cross-database analysis

**DOI:** 10.1186/s41065-021-00181-1

**Published:** 2021-04-23

**Authors:** Wanli Yang, Wei Zhou, Xinhui Zhao, Xiaoqian Wang, Lili Duan, Yiding Li, Liaoran Niu, Junfeng Chen, Yujie Zhang, Yu Han, Daiming Fan, Liu Hong

**Affiliations:** 1 State Key Laboratory of Cancer Biology and National Clinical Research Center for Digestive Diseases, Xijing Hospital of Digestive Diseases, Fourth Military Medical University, No.127, Changle West Road, Xi’an, 710032 Shaanxi Province China; 2 Department of Thyroid and Breast Surgery, The Affiliated Hospital of Northwest University & Xi’an No.3 Hospital, Northwest University, Xi’an, 710018 Shaanxi Province China; 3 Department of Otolaryngology, Xijing Hospital, Fourth Military Medical University, No.127, Changle West Road, Xi’an, 710032 Shaanxi Province China

**Keywords:** Oral squamous cell carcinoma, Bioinformatics analysis, Differentially expressed genes, GEO, TCGA

## Abstract

**Background:**

Oral squamous cell carcinoma (OSCC) is a malignant cancer, the survival rate of patients is disappointing. Therefore, it is necessary to identify the driven-genes and prognostic biomarkers in OSCC.

**Methods:**

Four Gene Expression Omnibus (GEO) datasets were integratedly analyzed using bioinformatics approaches, including identification of differentially expressed genes (DEGs), GO and KEGG analysis, construction of protein-protein interaction (PPI) network, selection of hub genes, analysis of prognostic information and genetic alterations of hub genes. ONCOMINE, The Cancer Genome Atlas (TCGA) and Human Protein Atlas databases were used to evaluate the expression and prognostic value of hub genes. Tumor immunity was assessed to investigate the functions of hub genes. Finally, Cox regression model was performed to construct a multiple-gene prognostic signature.

**Results:**

Totally 261 genes were found to be dysregulated. 10 genes were considered to be the hub genes. The Kaplan-Meier analysis showed that upregulated SPP1, FN1, CXCL8, BIRC5, PLAUR, and AURKA were related to poor outcomes in OSCC patients. FOXM1 and TPX2 were considered as the potential immunotherapeutic targets with future clinical significance. Moreover, we constructed a nine-gene signature (TEX101, DSG2, SCG5, ADA, BOC, SCARA5, FST, SOCS1, and STC2), which can be utilized to predict prognosis of OSCC patients effectively.

**Conclusion:**

These findings may provide new clues for exploring the molecular mechanisms and targeted therapy in OSCC. The hub genes and risk gene signature are helpful to the personalized treatment and prognostic judgement.

**Supplementary Information:**

The online version contains supplementary material available at 10.1186/s41065-021-00181-1.

## Background

Oral squamous cell carcinoma (OSCC) is the major type of head and neck squamous cell carcinoma (HNSCC) [1]. Despite great works have been made on early screen and personalized treatment for cancers, OSCC still is a challengeable disease and has brought seriously economic and medical burden [2]. Risk factors, including smoking, drinking, and HPV infections, are closely associated with the development of OSCC [3–5]. Nevertheless, the mechanisms of OSCC are still unclear. Moreover, most OSCC patients can’t be screened early due to the lack of available diagnostic markers. In addition, due to the drug resistance, some patients with OSCC might suffer from cancer recurrence. Thus, identifying the novel biomarkers and effective targets is of great importance to OSCC research and management.

Recently, the gene chip assays and second-generation gene sequencing have been extensively applied in scientific researches [6, 7]. These approaches could effectively screen the key genes that influence the cancer development or progression [8]. To date, numerous studies have used the second-generation gene sequencing or gene chip assays to explore the key genes in OSCC [9]. However, the findings might be inconsistent due to the tumor heterogeneity, localization, HPV-related link, and microbiota. So far, there are few reliable markers and therapeutic targets for OSCC. The integrated bioinformatics analysis may solve these problems and eventually find more convincing results since it uses several bioinformatics methods and integrates the data from different gene profiles [10].

In this study, we utilized microarray data of OSCC tissues and normal oral tissues in GEO and TCGA databases to screen the hub genes and biomarkers. We purposed to screen the hub genes, important GO terms, and significant pathways in the OSCC progression, thus helping to reveal the mechanisms of this disease. We also hope to pick out the risk genes and construct a multiple-gene prognostic signature, which can be implemented for prognostic judgment in OSCC.

## Material and methods

### Data collection and procession

To acquire the OSCC mRNA expression datasets, the keywords: “Expression profiling by array” “Homo sapiens”, and “Oral squamous cell carcinoma”, were searched in GEO database (http://www.ncbi.nlm.nih.gov/geo/) [11, 12]. After a systematic review, OSCC and non-tumor oral tissues gene expression profiles of GSE23558 [13], GSE30784 [14], GSE74530 [15], and GSE37991 [16] were selected and downloaded through getGEO function in “GEOquery” package [17]. The criteria for selecting the datasets as following: (1) dataset contains both OSCC tissues and non-tumor tissues; (2) samples size was 10 or more. The data were pre-processed as the previous studies [18, 19]. Briefly, the raw data of gene chips were normalized by “limma” package in R software (Version: 3.6.3). Moreover, the “sva” package was utilized to remove the batch effect. The detail information of the four GEO datasets was presented in Table 1. The RNA-seq data and clinicopathological data of 502 OSCC patients and 44 normal samples were also downloaded from The Cancer Genome Atlas (TCGA, https://cancergenome.nih.gov/) database. Data procession of TCGA database was performed as the previous studies [20, 21].

**Table 1:** The detail information of four GEO datasets

ID	Tissues	Platform	Normal(cases)	Tumor(cases)
GSE23558	OSCC	GPL6480	4	27
GSE30784	OSCC	GPL570	45	167
GSE74530	OSCC	GPL570	6	6
GSE37991	OSCC	GPL6883	40	40

*GEO* Gene Expression Omnibus, *OSCC* Oral squamous cell carcinoma

### Identification of DEGs

The limma package was utilized to screen the DEGs between OSCC and normal tissues in GEO datasets and TCGA dataset [22]. For selecting the DEGs in each GEO dataset, |logFC| >1 and adjust *P*-value <0.05 were set as the cut-off criteria [5]. Then, the overlapping DEGs was screened using a Venn tool (http://bioinfogp.cnb.csic.es/tools/venny/). The data profile of GSE37991 was used as a reference to construct the heatmap and to present the distribution of DEGs.

### Functional enrichment analysis for DEGs

GO and KEGG analysis of overlapping DEGs were conducted via the DAVID database (https://david.ncifcrf.gov/), and FunRich tool (FunRich 3.0) as descried previously [12, 23–25]. We submitted the overlapping DEGs into the above databases or software. The top five GO terms for biological process (BP), cellular component (CC), and molecular function (MF) were illustrated as bar charts [12]. The KEGG results were visualized by “clusterProfiler” package, the top 10 KEGG pathways of upregulated DEGs and downregulated DEGs were showed as bubble charts, respectively [26].

### Construction of protein-protein interaction (PPI) network

PPI networks are formed by proteins due to the existence of biochemical or electrostatic forces [27]. Here, the Search Tool for the Retrieval of Interacting Genes (STRING 11.0) database (https://string-db.org/cgi/input.pl) was applied to establish PPI networks [12, 27]. Cytoscape software (v3.6.1) was used to illustrate the PPI networks and the cut-off criteria were set as confidence score ≥ 0.4, maximum number of interactors = 0 [12, 28]. Gene clusters visualization are conducted as our previous studies [12, 28]. Gene clusters were screened with the following criteria: MCODE scores >10 and number of nodes >10 [12]. 10 genes were considered to be the hub genes according to connectivity degree [12, 28, 29].

### Hub genes validation

As for hub genes validation, we assessed the levels both in mRNA level and protein level. Specifically, ONCOMINE (https://www.oncomine.org) database was utilized to evaluate the mRNA expression of selected hub genes [30]. The gene rank means that the median rank of one searched gene across the selected analyses [31]. We also used GEPIA database(http://gepia.cancer-pku.cn/index.html) to validate the mRNA levels of hub genes [12, 32]. The Human Protein Atlas database collected more than 11,200 unique proteins [33], we thus used it to evaluate the protein levels of hub genes in OSCC tissues and normal control tissues.

### Genetic alterations and survival analysis

The cBio Cancer Genomics Portal (http://www.cbioportal.org/) is an online database which enables us to compare the genetic alterations of the hub genes in OSCC [34]. Subsequently, survival analysis was performed in the Kaplan Meier-plotter website (http://kmplot.com/analysis/index.php), which could assess the effect of 54,675 genes on survival using 18,674 cancer samples from GEO, European Genome-phenome Archive (EGA) and TCGA database [35].

### Analysis of potential target genes

TIMER website (https://cistrome.shinyapps.io/timer/) was applied to investigate the expression levels of the selected target genes in different human tumors [36]. The UALCAN website (http://ualcan.path.uab.edu/analysis.html) was utilized to assess the factors which are associated with the expression levels of the target genes in OSCC [37].

### Correlation analysis of genes expression and immune cell infiltration and immune checkpoints

TIMER website was applied to analyze the genes expression data for FOXM1 and TPX2 in TCGA OSCC samples and its correlation with tumor infiltration of six immune cell types (B cells, CD4+T cells, CD8+ T cells, neutrophils, macrophages, and dendritic cells) and five immunological checkpoints (CD274, CTLA4, PDCD1, PDCD1LG2, and TOX) [36].

### Construction of prognostic gene signature

Univariate and multivariate Cox regression analyses were conducted to investigate the relationships between the expression of 261 overlapping DEGs and OSCC patients’ survival in TCGA dataset. Nine DEGs were identified as the prognostic indicators in OSCC. These nine DEGs were utilized to construct a prognostic signature as previous study [38]. Each OSCC patient received a risk score according to the formula: risk score = (Coefficient gene 1 × Expression gene 1) + (Coefficient gene 2 × Expression gene 2) +…+ (Expression gene n × Coefficient gene n). Finally, the Kaplan-Meier curve and receiver operating characteristic (ROC) curve were used to evaluate the efficiency of the gene signature.

### Identification of the independent prognostic indicators in OSCC

Univariate and multivariate Cox regression analyses were conducted to identify the independent prognostic indicators (including age, gender, grade, stage, T, M, N, and risk score) for OSCC patients.

### Statistical analysis

The R statistical package (R version 3.6.3) and Perl language and were utilized to conduct the statistical tests and graphics unless otherwise stated. P <0.05 was regarded as statistical significance.

## Results

### DEGs involved in OSCC

**Fig. 1:**
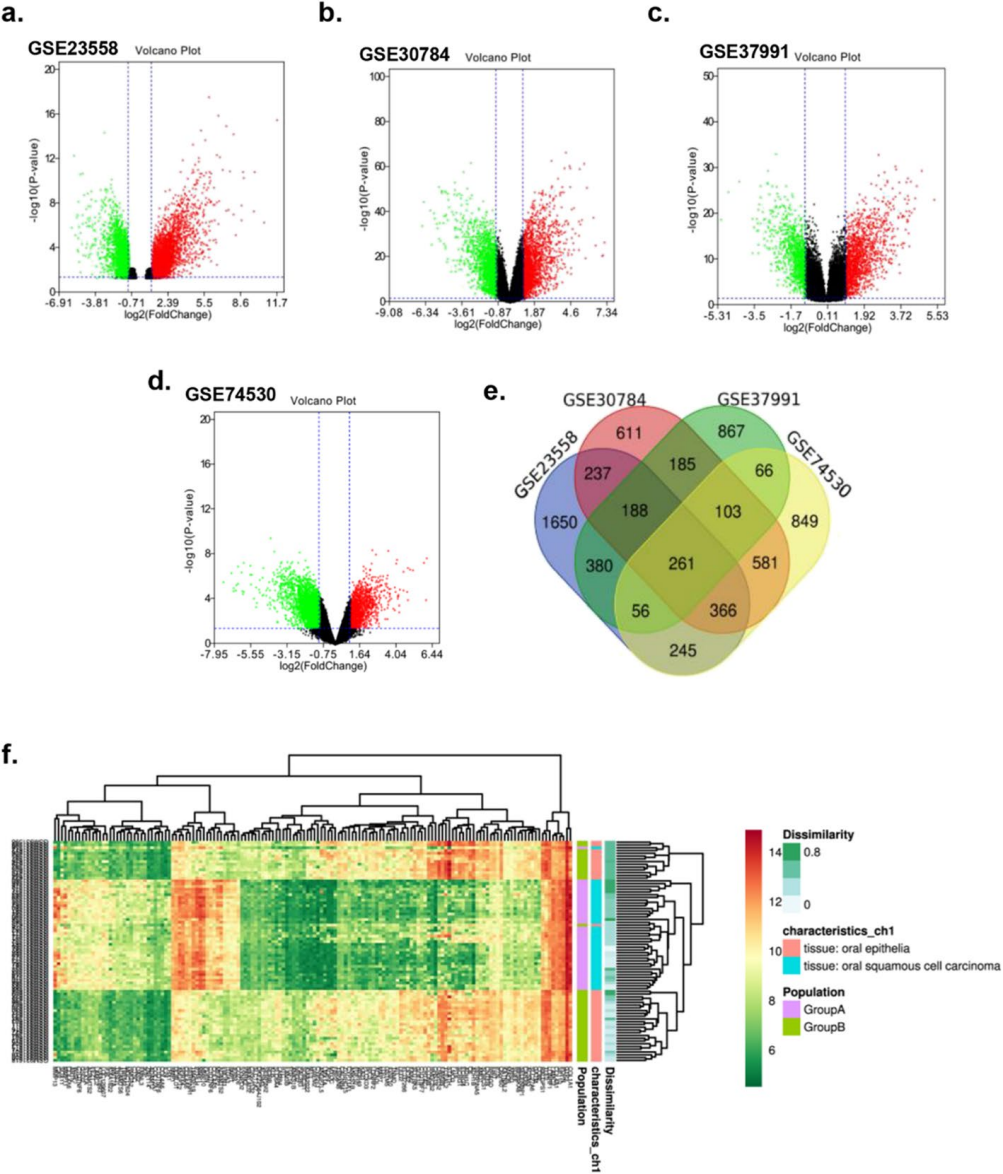
Identification of 261 DEGs from the four profile datasets. **a** Volcano plot of the GSE23558; **b** Volcano plot of the GSE30784; **c** Volcano plot of the GSE37991; **d** Volcano plot of the GSE74530 (red color dots represent the upregulated genes; green color dots represent the downregulated genes); **e** Venn diagram was utilized to screen the overlapping DEGs in four datasets. **f** The data profile of GSE37991 was used as a reference to construct the heatmap and to show the differential distribution of DEGs. DEGs, differentially expressed genes; logFC, log‑fold change

The four datasets (GSE23558, GSE30784, GSE74530, and GSE37991), including 240 OSCC tissues and 95 non-tumor tissues, were included in this study. We extracted 3383, 2532, 2106, and 2527 DEGs from GSE23588, GSE30784, GSE37991, and GSE74530, respectively (Fig. 1a-d). Totally 261 overlapping DEGs were screened from the 4 datasets (Fig. 1e; Table 2), including 135 upregulated DEGs and 126 downregulated DEGs. The data profile of GSE37991 was used as a reference to construct the heatmap and to show the differential distribution of DEGs (Fig. 1f).

**Table 2:** 261 overlapping DEGs in OSCC of four gene expression profiles

DEGs	Gene names
Upregulated DEGs	BAX, NEK6, SSH1, SLC3A2, EPHB2, TNFRSF10B, STX2, IKBIP, IGF1R, ADA, MARCKSL1, PDLIM7, BIRC5, COL4A5, CARD10, CTSC, MCM2, BNC1, ABL2, ARPC1B, HLA-F, TGIF1, FBLIM1, COLGALT1, CDCA8, GNA12, HAPLN3, BMP1, TRIO, DNMT3B, SOCS1, NRP2, PTK7, COL4A1, HOMER3, PDE7A, SLC28A3, ANGPT2, SERPINH1, NLRC5, FXYD5, MB21D1, MCM4, LHFPL2, ADAM12, TK1, DCBLD1, PLAUR, SPRY4, COL12A1, PML, EXO1, APOL1, HAS3, CDCA3, AGRN, RTKN, TEAD4, CHEK1, ITGA5, TMEM132A, CD276, HSD17B6, STC2, LTBP1, SHCBP1, SCD5, UBE2L6, SNX10, LAMA3, CXCL5, SP110, TPX2, FOXD1, LPAR3, HOXD10, CD274, TENM2, ITGB4, FOXM1, DSG2, AURKA, OAS2, MELK, E2F7, CDC6, KIF23, NRG1, FEZ1, CTSL, PLEK2, CDH3, AIM2, TRIP13, COL4A6, ADTRP, PCDH7, BST2, OASL, LAMC2, IL11, FN1, SOX11, PLAU, ITGA3, EPSTI1, SERPINE1, WDR66, TNFRSF12A, PDPN, IFIT3, LY6K, IFI6, GALNT6, ISG15, IL1RL1, CXCL8, HMGA2, CXCL9, RSAD2, SCG5, CYP27B1, GBP5, ZNF114, IL24, FST, PTHLH, NELL2, INHBA, SLCO1B3, SPP1, CXCL10, MMP1, MMP10, MMP3
Downregulated DEGs	TMPRSS11B, MAL, CRNN, FAM3B, TYRP1, ALOX12, KRT4, AADAC, CLDN17, CTTNBP2, PPP1R3C, KRT13, ENDOU, OGN, HLF, COBL, CILP CLCA4 SH3GL3, MFAP4, ALDH3A1, ABCA8, CYP4B1, FUT6, ATP13A4, FAM3D, SH3BGRL2, SCNN1B, ANKRD6, RRAGD, GATM, HOPX, KLB, ASPA, CYP2C18, MUC15, OCLN, PTN, LAMB4, C2orf54 WNK4, CXCR2, ANGPTL1, ACPP, PBX1, SCARA5, UPK1A, TSPAN8, GALNT5, SLC6A4, BEX4, ATP6V0A4, CYP2C9, GULP1, CXCL17, SUSD4, SYTL4, MLPH, APOD, MAOB, MIR99AHG, DPT, NEBL, SLURP1, ECHDC3, TEX101, SAMD5, RBP7, FAM189A2, FAM221A, GALNT12, GYS2, LRRK2, AR, SYNGR1, SFTA2, MYZAP, GGTA1P, C15orf59, GAS7, DEPTOR, CYP4F12, FUT3, KIAA1211L, BOC, PAX9, MAMDC2, PPARGC1A, CEACAM1, RNASE4, CYP2J2, TGFBR3, CXCL13, RALGPS1, GKAP1, EPHX2, DCT, SLC16A7, IL1RN, PSCA, BCAS1, SPATA18, CFD, PLAGL1, CD207, SORBS1, MGLL, SPAG16, GREM2, GSTM5, SLC4A4, SCIN, CAB39L, CYSLTR1, ACADSB, SHROOM3, PLIN1, GGT6, PAQR8, GPD1L, PGD, PANK1, ATP6V1C2, ITM2C, ABCA5, ZNF273

*DEGs* differentially expressed genes, *OSCC* Oral squamous cell carcinoma

### Functional enrichment analysis

Functional enrichment analysis was analyzed by DAVID database and FunRich tool (FunRich 3.0). As shown in Figure S1a, in BP group, upregulated DEGs were mainly involved in energy pathways, metabolism, and lipid storage, whereas the downregulated DEGs were related to signal transduction, cell communication, and immune response (Figure S1b). For CC group, upregulated DEGs were mainly associated with exosomes, extracellular space, and endoplasmic reticulum membrane, whereas downregulated DEGs were involved in extracellular, extracellular region, and extracellular space. As for MF, upregulated DEGs were correlated to fucosyltransferase activity, catalytic activity, and cytokine activity. The downregulated DEGs were significantly connected with to extracellular matrix structural constituent, metallopeptidase activity, and cytokine activity. According to the GO results, subsequent studies on OSCC should focus on how to balance the cellular microenvironment or reshape the cellular physiological functions of cancer cells.

KEGG enrichment showed that upregulated DEGs were mainly involved in pathway in cancer, PI3K-Akt pathway, ECM receptor interaction, and focal adhesion (Figure S1c). Previous studies have showed that molecular pathways involved in OSCC are complex [39]. The PI3K-Akt and Wnt/β-catenin signaling were demonstrated to be the three major interlinked pathways involved in the molecular pathogenesis of OSCC [39]. Here, we found that pathway in cancer and PI3K-Akt pathway were associated with the upregulated DEGs. Therefore, monitoring these signaling pathways may aid to decide appropriate therapeutic approaches in OSCC patients. The downregulated DEGs were enriched metabolic pathway, serotonergic synapse, tyrosine metabolism, and arachidonic acid metabolism (Figure S1d). Studies have showed that metabolic alterations might provide energy and nutrients for sustaining the cancer proliferation and growth [40]. In this study, downregulated DEGs were closely related to metabolic pathway, tyrosine metabolism, and arachidonic acid metabolism. Therefore, these metabolism-targeted pathways may help us to improve the treatments efficiency.

### PPI network analysis

**Fig. 2:**
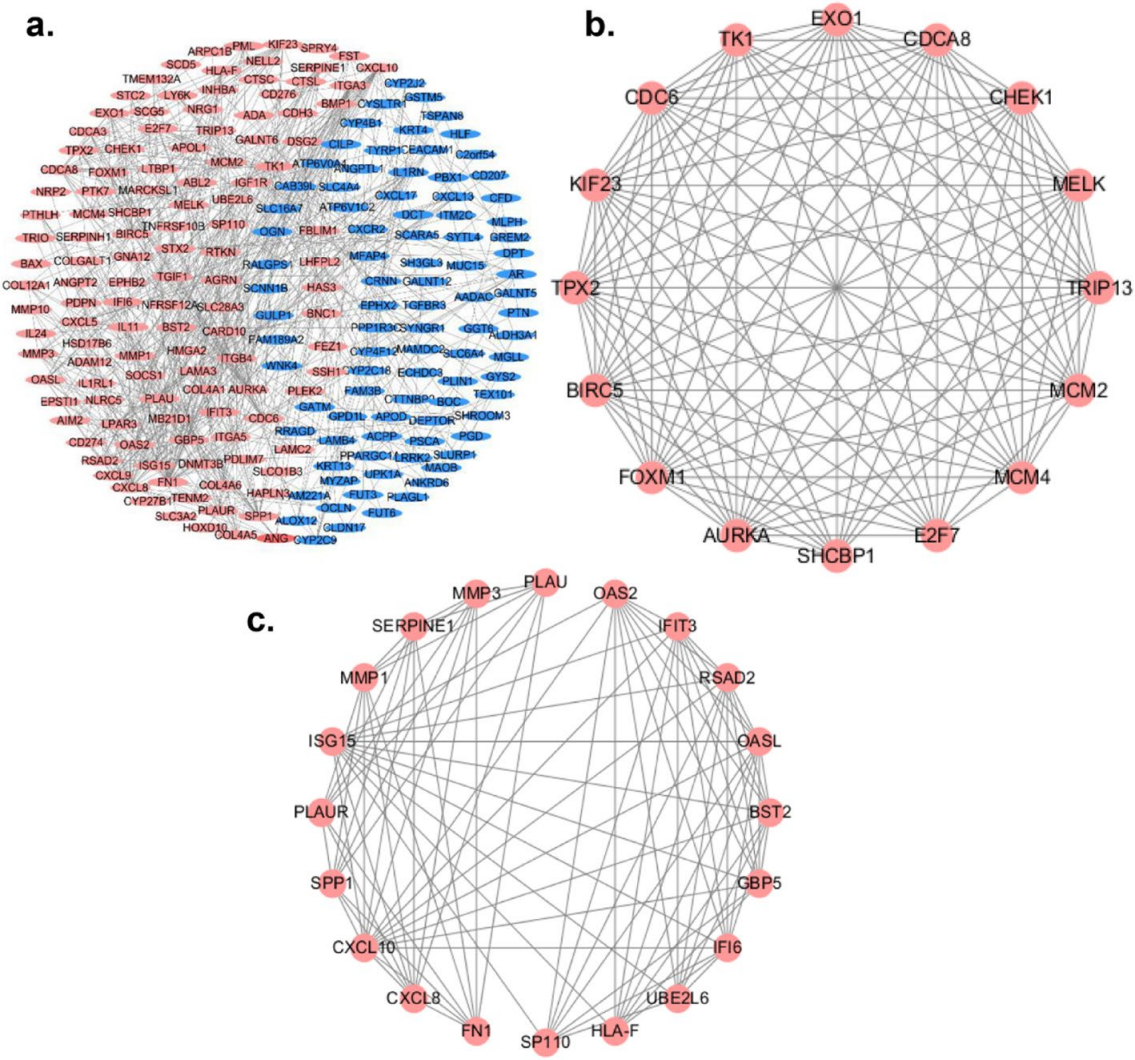
Construction and analysis of PPI network of DEGs. **a** PPI was established by the 261 overlapping DEGs using STRING database; **b** Cluster 1; **c** Cluster 2; Upregulated DEGs (red) or downregulated DEGs (blue) is indicated. PPI, protein-protein interaction; DEGs, differentially expressed genes; STRING, search tool for the retrieval of interacting genes

Totally 261 overlapping DEGs were mapped into PPI network via STRING database and Cytoscape software, (Fig. 2a). The top two important clusters were picked out by MCODE plug-in in Cytoscape software (Cluster 1, MCODE score =15.2; Cluster 2, MCODE score = 9.789) (Fig. 2b-c). Cluster 1 consists of 16 nodes and 144 edges (Table S1), which are mainly related to cell cycle, DNA replication, and cellular senescence. Cluster 2 consists of 20 nodes and 93 edges (Table S2), which are mainly enriched in IL-17 signaling pathway, influenza A, and complement and coagulation cascades. Using cytoHubba software, 10 genes (FN1, CXCL8, CXCL10, SPP1, FOXM1, AURKA, ISG15, PLAUR, TPX2, and BIRC5) were selected as hub genes according to the connectivity degree (Table 3). According to Figure S2a, the hub genes could interact with each other and they might be the driven-genes in OSCC development and progression. KEGG pathways analysis showed that the significantly enriched terms for the hub genes were RIG-I-like receptor signaling pathway, Toll-like receptor signaling pathway, IL-17 signaling pathway, Influenza A, Cellular senescence, Chemokine signaling pathway, and Cytokine-cytokine receptor interaction (Figure S2b).

**Table 3:** Top 10 hub genes with higher degree of connectivity

Genes	Gene description	Degree of connectivity	Betweenness
FN1	Fibronectin 1	51	14204.03675
CXCL8	C-X-C motif chemokine ligand 8	32	4144.65638
CXCL10	C-X-C motif chemokine ligand 10	28	3898.53933
SPP1	Secreted phosphoprotein 1	21	2115.86473
FOXM1	Forkhead box M1	20	1391.36367
AURKA	Aurora kinase A	20	2706.35927
ISG15	ISG15 ubiquitin like modifier	19	1081.35875
PLAUR	Plasminogen activator, urokinase receptor	19	3931.73348
TPX2	TPX2 microtubule nucleation factor	18	266.62393
BIRC5	Baculoviral IAP repeat containing 5	18	1221.29117

*DEGs* differentially expressed genes, *OSCC* Oral squamous cell carcinoma

### Validation the expression of hub genes

**Fig. 3:**
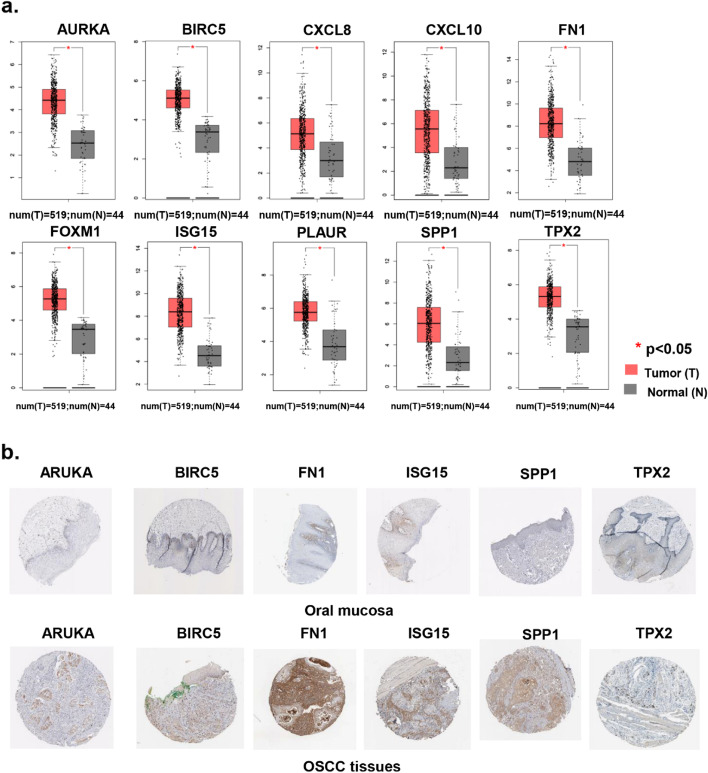
Validation of hub genes in TCGA database and Human Protein Atlas database. **a** Box plots show the mRNA levels of the 10 hub genes (FN1, CXCL8, CXCL10, SPP1, FOXM1, AURKA, ISG15, PLAUR, TPX2, and BIRC5) in HNSCC/OSCC tissues and normal oral tissues using data from the TCGA database in GEPIA (http://gepia.cancer-pku.cn/index.html). The validation results were consistent with these observed in GEO datasets. *P < 0.05 was considered statistically significant. **b** Representative immunohistochemistry images of AURKA, BIRC5, FN1, ISG15, SPP1, TPX2 in OSCC and normal oral tissues derived from the Human Protein Atlas database; OSCC, Oral squamous cell carcinoma; HNSCC, Head and neck squamous cell carcinoma; TCGA, The Cancer Genome Atlas

ONCOMINE, TCGA, and The Human Protein Atlas databases were used to validate the expression of hub genes. Firstly, ONCOMINE was used to perform a meta‑analysis to compare the mRNA levels of FN1, CXCL8, CXCL10, SPP1, FOXM1, AURKA, ISG15, PLAUR, TPX2, and BIRC5 between OSCC and non-tumor oral tissues. As showed in Figure S3, the mRNA expression levels of FN1 (Figure S3a), CXCL8 (Figure S3b), CXCL10 (Figure S3c), SPP1 (Figure S3d), FOXM1 (Figure S3e), AURKA (Figure S3f), ISG15 (Figure S3g), PLAUR (Figure S3h), TPX2 (Figure S3i), and BIRC5 (Figure S3j) were significantly upregulated in OSCC tissues compared to those in normal oral tissues (*P *< 0.05). In addition, the median rank of FOXM1 was the highest among the top 10 hub genes in OSCC tissues (Figure S3e). The findings from GEPIA database also demonstrated that the 10 hub genes were significantly higher in OSCC tissues than those of non-tumor tissues (Fig. 3a). These results were consistent with the observed in GEO datasets. Due to the lack of CXCL8, CXCL10, FOXM1 and PLAUR information in The Human Protein Atlas dataset, their protein expression level was not analyzed (Fig. 3b). The results indicated that the protein expressions of AURKA, BIRC5, FN1, ISG15, SPP1 and TPX2 were overexpressed in OSCC compared with the control samples (Fig. 3b).

### Genetic alterations and survival analysis of hub genes

**Fig. 4:**
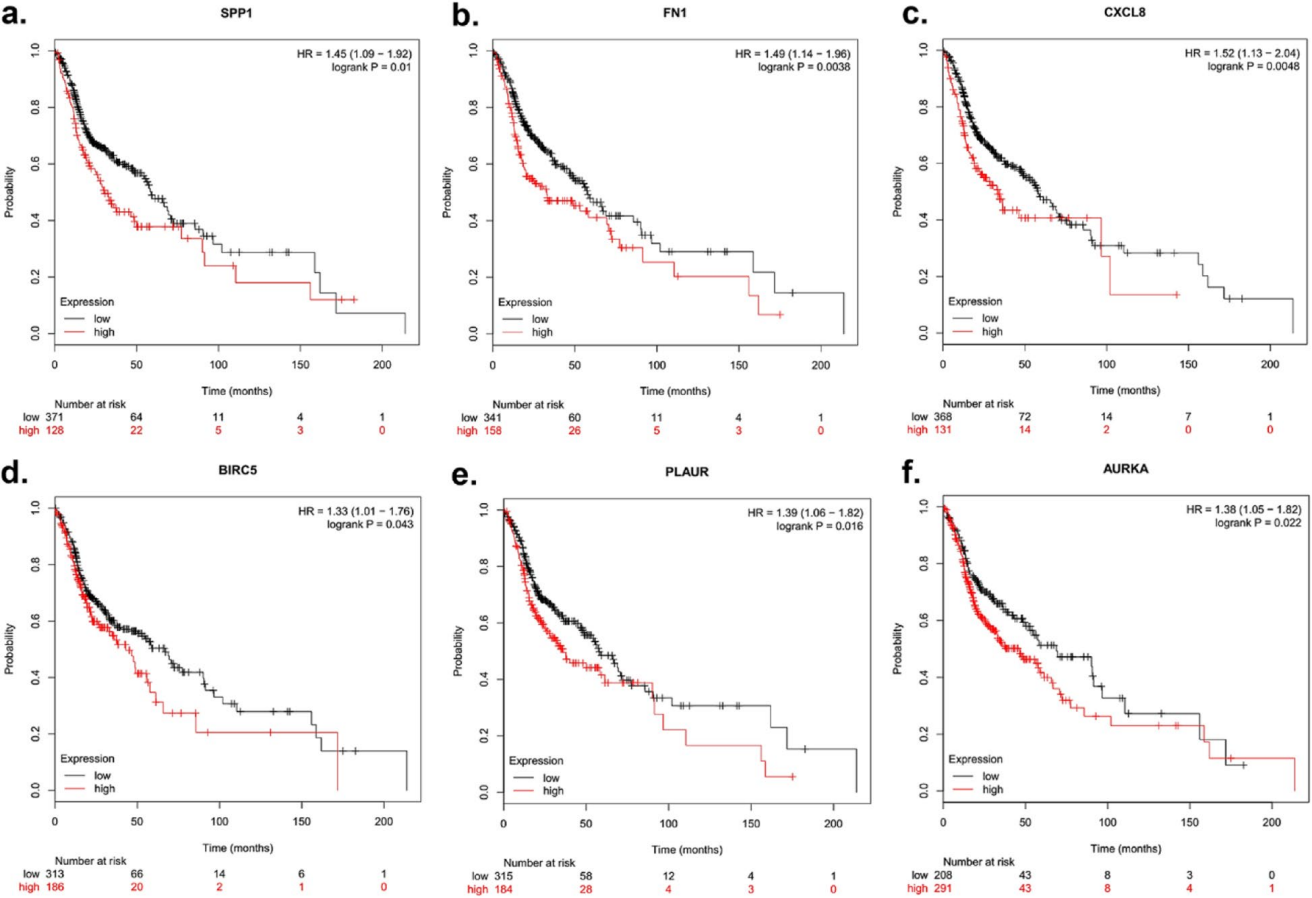
OS of the 10 hub genes in OSCC patients was analyzed via Kaplan‑Meier plotter. Prognostic values of (**a**) SPP1, (**b**) FN1, (**c**) CXCL8, (**d**) BIRC5, (**e**) PLAUR, and (**f**) AURKA were obtained in the Kaplan Meier-plotter website (http://kmplot.com/analysis/index.php). Data are shown as the hazard ratio with 95% confidence interval. The red plots present the high expression of each patients while the black plots present the low expression of each patients. OSCC, Oral squamous cell carcinoma; OS, Overall survival

**Fig. 5:**
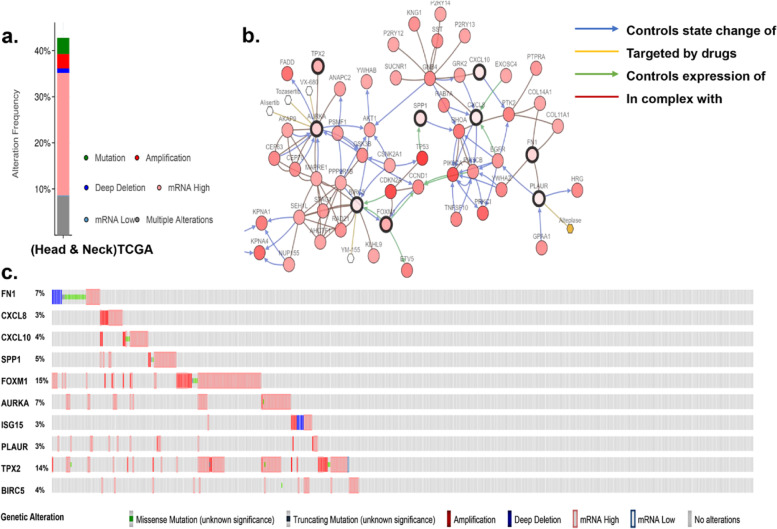
Genetic alterations and therapeutic potential of hub genes. **a** An overview of genetic changes of 10 hub genes in TCGA; **b** A visual summary across a set of OSCC shows the genetic alterations connected with the 10 hub genes which were altered in 217(44%) of 496 sequenced cases/patients (496 total); **c** The network includes 57 nodes (10 hub genes and their 47 neighbor genes). The drugs for these 10 hub genes are also presented. OSCC, Oral squamous cell carcinoma; TCGA, The Cancer Genome Atlas

Kaplan Meier-plotter website was applied to evaluate the prognostic potential of the 10 hub genes. The results demonstrated that high levels of SPP1 (HR=1.45(1.09-1.92), P=0.01), FN1 (HR=1.49(1.14-1.96), P=0.0038), CXCL8 (HR=1.52(1.13-2.04), P=0.0048), BIRC5 (HR=1.33(1.01-1.76), P=0.043), PLAUR (HR=1.39(1.06-1.82), P=0.016), and AURKA (HR=1.44(1.05-18.2), P=0.022) were associated with poor OS in OSCC patients (Fig. 4). The remaining four genes present similar trends, but not statistically significant. Moreover, the genetic alterations were enquired by cBioPortal. Figure 5a and b showed the alteration state of 10 genes. These 10 genes were changed in 217 (44%) of 496 sequenced patients (496 total), and that FOXM1 and TPX2 were changed most often (15% and 14%), including amplification, missense mutation, and deep deletion. Figure 5c illustrated the network established by the 10 hub genes and their 47 neighbor genes. Besides, drugs targeting the hub genes were presented. According to Fig. 5c, only AURKA, BIRC5, and PLAUR were utilized as chemotherapy targets for cancer treatment presently. Therefore, we supposed that the other seven genes (FN1, CXCL8, CXCL10, SPP1, FOXM1, ISG15, and TPX2) might be the novel targets for OSCC treatment.

### Biological functions of FOXM1 and TPX2 in tumors

**Fig. 6:**
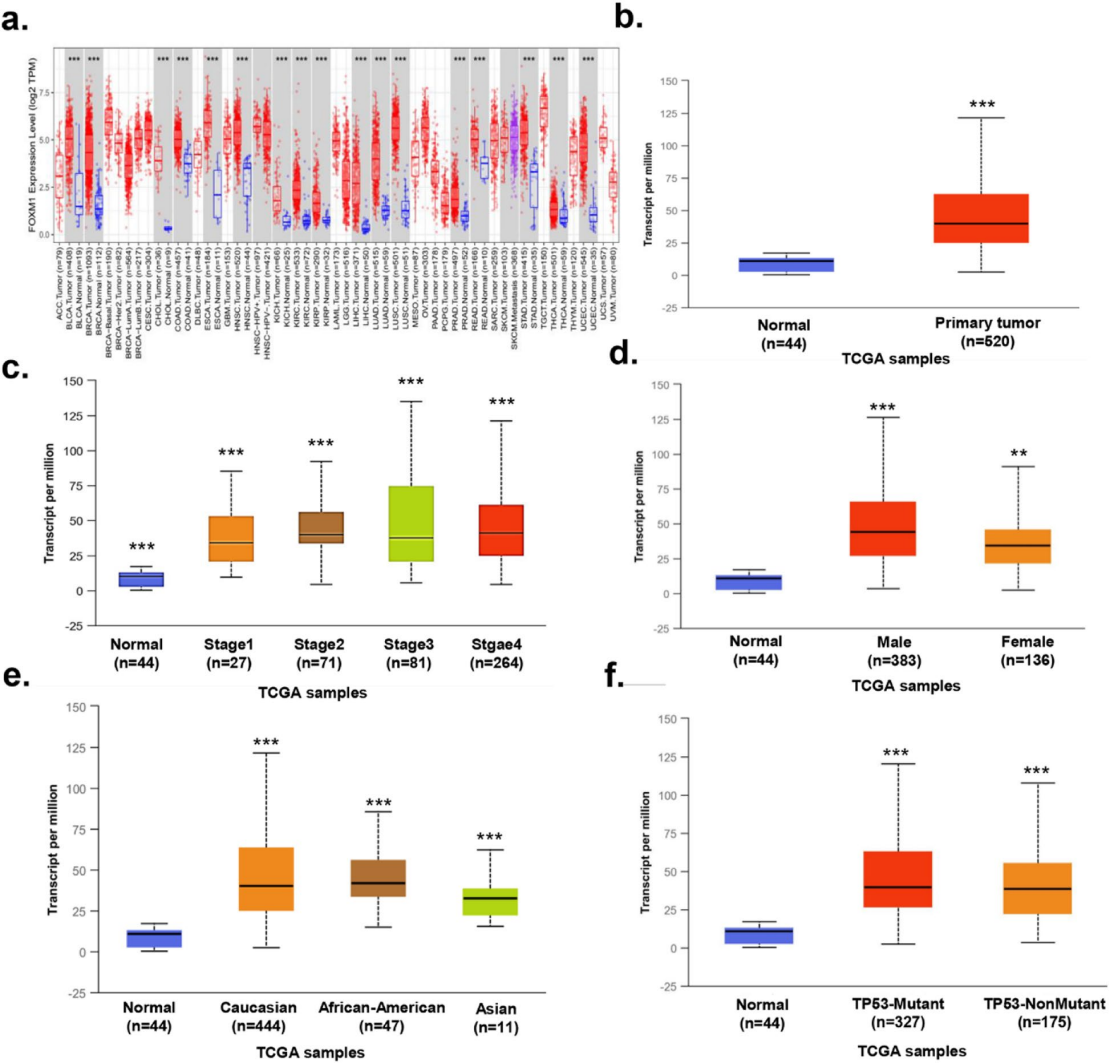
Biological function of FOXM1 in tumors. **a** Expression of FOXM1 in various tumors. **b** Expression of FOXM1 in OSCC tissues and normal controls. **c** Expression of FOXM1 in OSCC based on tumor stages. **d** Expression of FOXM1 in OSCC based on genders. **e** Expression of FOXM1 in OSCC based on races. **f** Expression of FOXM1 in OSCC based on molecular subtypes of OSCC. ∗*P *< 0.05, ∗∗*P* < 0.01, ∗∗∗*P* < 0.001. OSCC, Oral squamous cell carcinoma; FOXM1, Forkhead box protein M1

**Fig. 7:**
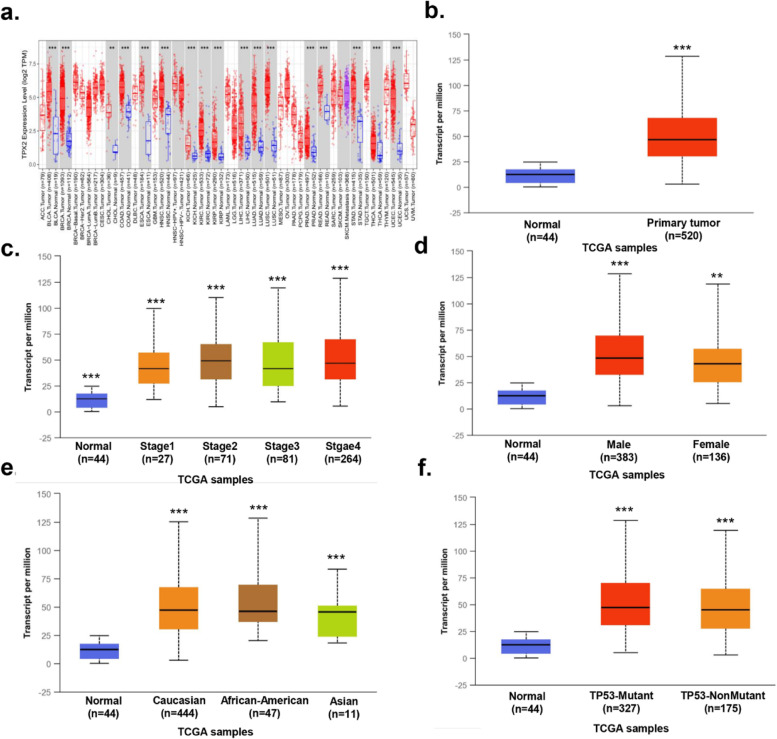
Biological function of TPX2 in tumors. **a** Expression of TPX2 in various tumors. **b** Expression of TPX2 in OSCC tissues and normal controls. **c** Expression of TPX2 in OSCC based on tumor stages. **d** Expression of TPX2 in OSCC based on genders. **e** Expression of TPX2 in OSCC based on races. **f** Expression of TPX2 in OSCC based on molecular subtypes of OSCC. ∗*P* < 0.05, ∗∗*P* < 0.01, ∗∗∗*P* < 0.001. OSCC, Oral squamous cell carcinoma; TPX2, Targeting protein for xenopus kinesin-like protein 2

As FOXM1 and TPX2 were changed most often in OSCC, we thus chose FOXM1 and TPX2 as the target genes to conduct the following studies. Firstly, TIMER website was used to explore the expression levels of FOXM1 in several tumors and corresponding normal tissues. FOXM1 is upregulated in a variety of tumors, including BLCA, BRCA, CHOL, COAD, ESCA, HNSC, KICH, KIRC, KIRP, LIHC, LUAD, LUSC, PPAD, READ, STAD, THCA, and UCEC (Fig. 6a). This suggests that FOXM1 may also play an oncogenic role in other tumors. Moreover, by using the UALCAN website, we found that FOXM1 is highly expressed in OSCC tissues (Fig. 6b). FOXM1 has differential expression in patients with different tumor stages (Fig. 6c), genders (Fig. 6d), races (Fig. 6e), and molecular subtypes (Fig. 6f). Similarly, TPX2 is upregulated in a variety of human tumors, including BLCA, BRCA, CHOL, COAD, ESCA, HNSC, KICH, KIRC, KIRP, LIHC, LUAD, LUSC, PPAD, READ, STAD, THCA, and UCEC (Fig. 7a). We also found that TPX2 is highly expressed in OSCC tissues via the UALCAN website (Fig. 7b). In addition, TPX2 has differential expression in patients with different tumor stages (Fig. 7c), genders (Fig. 7d), races (Fig. 7e), and molecular subtypes (Fig. 7f).

### FOXM1 and TPX2 act as the immune-associated genes in OSCC

**Fig. 8:**
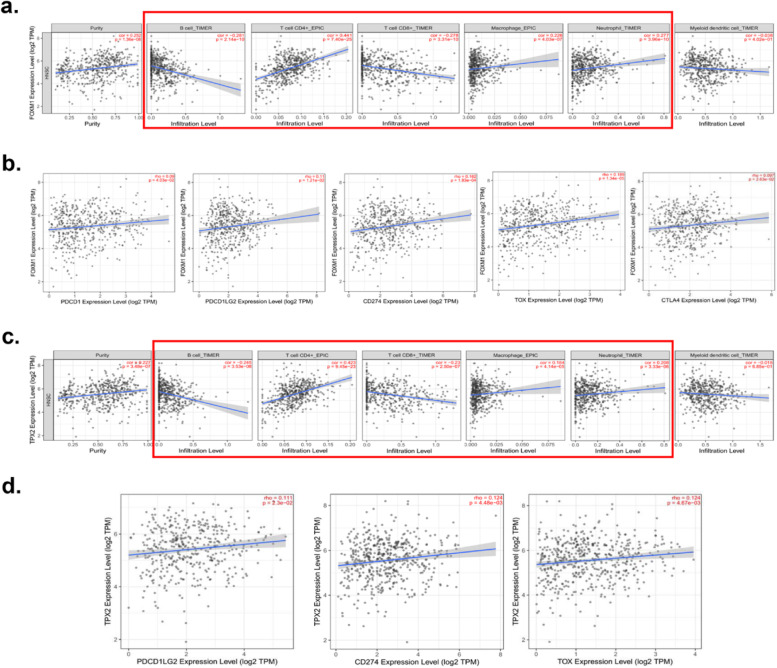
Tumor immune correlation analysis. **a** Relationship between FOXM1 expression and immune cells. **b** Relationship between FOXM1 expression and immune checkpoints; **c** Relationship between TPX2 expression and immune cells. **d** Relationship between TPX2 expression and immune checkpoints. FOXM1, Forkhead box protein M1; TPX2, Targeting protein for xenopus kinesin-like protein 2

The TIMER website was applied to analyze the relationship between FOXM1, TPX2, and immune cell infiltration. The results showed that both FOXM1 and TPX2 are involved in the infiltration of B cells, CD4+ T cells, CD8+ T cells, Macrophage cells and Neutrophils cells in OSCC (Fig. 8a, c). Moreover, we further analyzed the co-expression relationship of FOXM1, TPX2, and immune checkpoint-related genes CD274, CTLA4, PDCD1, PDCD1LG2, and TOX. Interesting, we found that FOXM1 was markedly correlated with PDCD1, PDCD1LG2, CD274, TOX, and CTLA4 (Fig. 8b). And TPX2 has a significant co-expression relationship with PDCD1LG2, CD274, and TOX (Fig. 8d). These findings suggest that FOXM1 and TPX2 may act as the immune-related therapeutic targets in OSCC.

### Construction of the risk gene signature for OSCC patients

To construct a promising risk gene signature using the 261 overlapping DEGs in OSCC, univariate analysis was performed to identify the DEGs related to the prognosis of OSCC patients using the TCGA dataset. Totally 42 overlapping DEGs was markedly associated with the prognosis of OSCC patients (Table S3). A stepwise multivariate Cox regression was then conducted to construct the risk gene signature. Nine candidate genes (TEX101, DSG2, SCG5, ADA, BOC, SCARA5, FST, SOCS1, and STC2) were identified as the significant prognostic indicators for OSCC patients (Table S4). Each OSCC patient was assigned a risk score calculated as follows: risk score = (-0.32654 × expression value of TEX101) + (0.10257 × expression value of DSG2) + (0.15022 × expression value of SCG5) + (0.18621 × expression value of ADA) + (-0.29365 × expression value of BOC) + (-0.26023 × expression value of SCARA5) + (0.10796 × expression value of FST) + (-0.26676 × expression value of SOCS1) + (0.15679 × expression value of STC2).

**Fig. 9:**
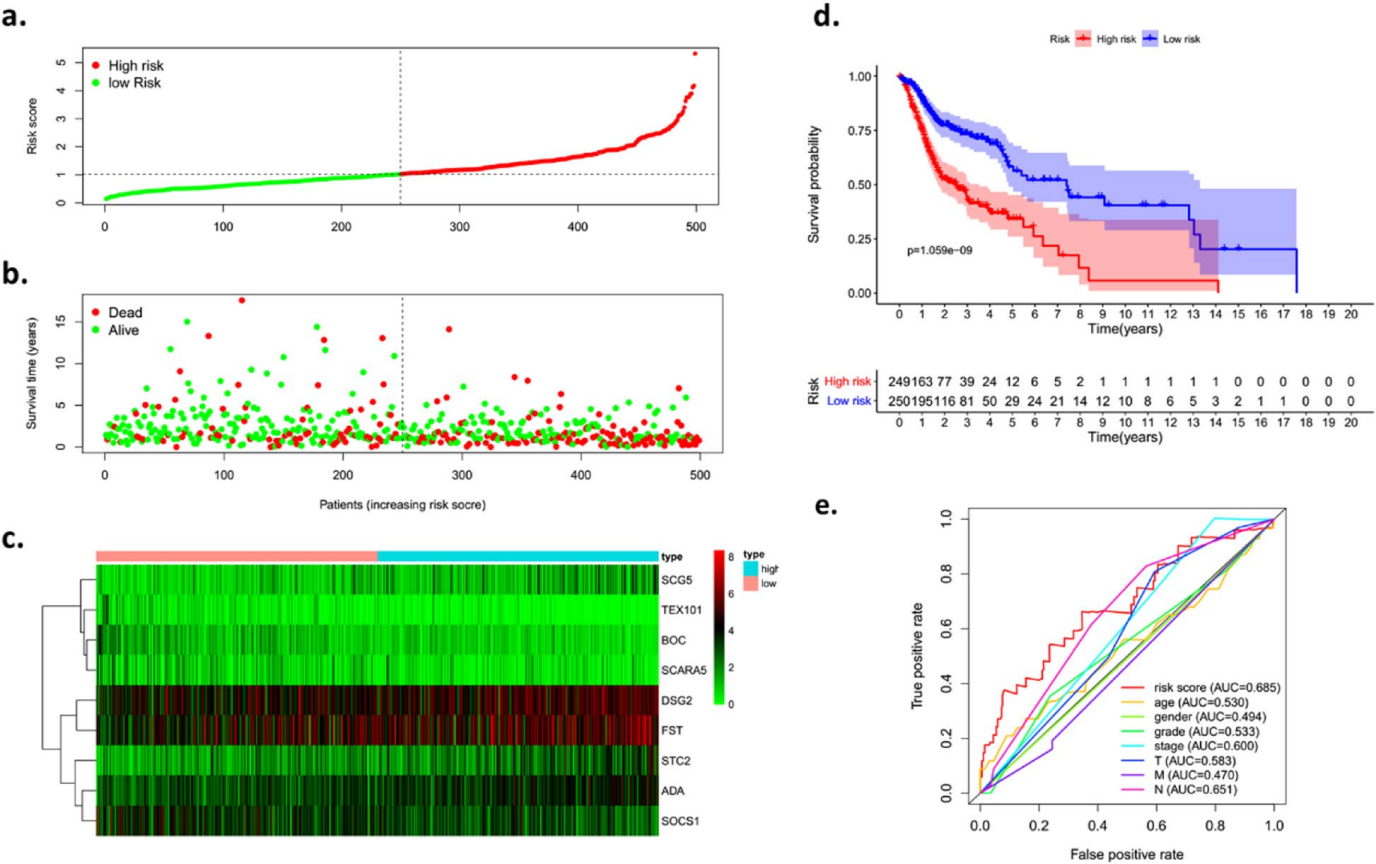
Construction of prognostic model for OSCC patents. **a** The distribution of risk scores of OSCC patients in prognostic model. **b** The distribution of OSCC patients with different survival status. **c** The heatmap of nine risk genes (TEX101, DSG2, SCG5, ADA, BOC, SCARA5, FST, SOCS1, and STC2) in OSCC patients in high-risk and low-risk group. **d** Kaplan-Meier curves of OSCC patients in high-risk and low-risk group. **e** The ROC curve for evaluating the performance of the prognostic model

The OSCC patients were divided into high-risk and low-risk group according to the median value of risk score (Fig. 9a-c). Figure 9a-b shows that the survival time of OSCC patients decreases along with the rising of risk score. As shown in Fig. 9d, the Kaplan-Meier curves shows that OSCC patients with lower risk score present a longer survival time than those with high risk score (P=1.059e-09). Figure 9e shows that the risk score curve presents a good feasibility in predicting the patients’ survival with AUC of 0.685.

### The risk score, stage and N were independent prognostic indicators in OSCC

**Fig. 10:**
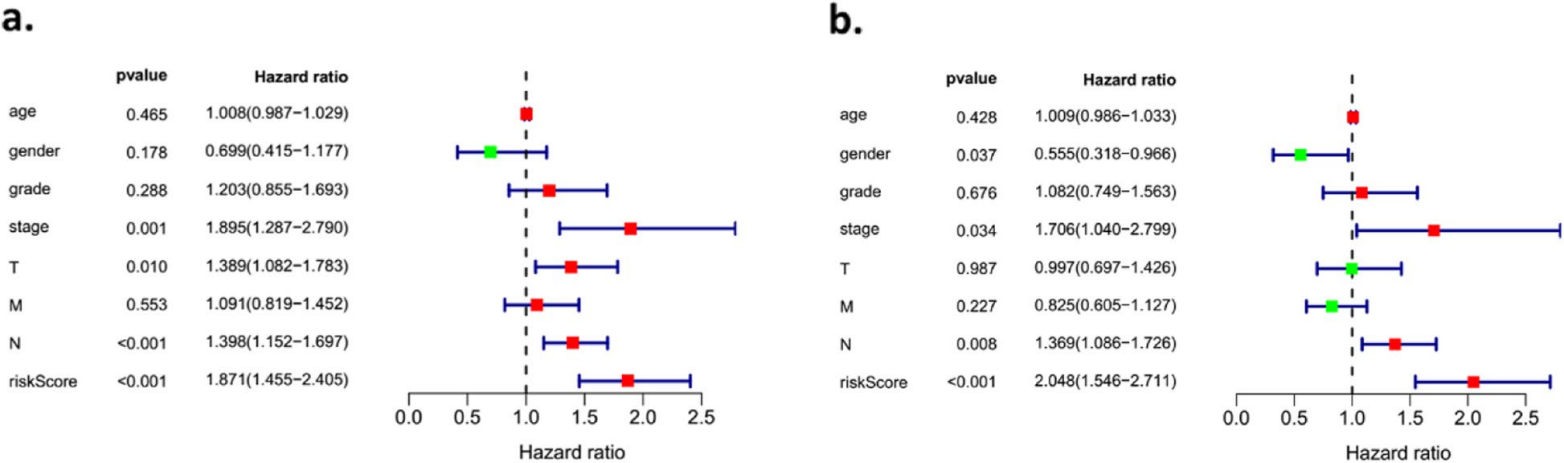
Independent prognostic indicators in OSCC. **a** Univariate Cox regression analysis of the clinicopathological characteristics and risk score in OSCC; **b** Multivariate Cox regression analysis of the clinicopathological factors and risk score in OSCC

As shown in Fig. 10a, the risk score was significantly associated with the poorer OS in OSCC (HR=1.871, 95% CI: 1.455-2.405, *P *< 0.001). Moreover, stage (HR=1.895, 95% CI: 1.287-2.790, *P *< 0.01), N (lymph nodes) (HR=1.398, 95% CI: 1.152-1.697, *P *< 0.001), and T (primary tumor) (HR=1.389, 95% CI: 1.082-1.783, *P* < 0.01) were also related to the OS. Then, all these factors were entered into multivariate Cox analysis. The risk score (HR=2.048, 95% CI: 1.546-2.711, *P* < 0.001), stage (HR=1.706, 95% CI: 1.040-2.799, *P* < 0.05), and N (HR=1.368, 95% CI: 1.086-1.726, *P* < 0.01) were still identified as the independent prognostic indicators for worse OS in OSCC patients (Fig. 10b).

## Discussion

Nowadays, the incidence rate of OSCC is still increasing quickly [41]. It is estimated that over 354,864 new cases and 177,384 deaths occurred in 2018 [41]. Compared with the other researches that only explored a single cohort or several genes, our study used several databases to integratedly investigate the key genes, pathways, biomarkers and risk gene signature in OSCC development. In this study, totally 261 overlapping DEGs (135 upregulated DEGs and 126 downregulated DEGs) were identified. KEGG pathway enrichment analysis shows that pathway in cancer, PI3K-Akt signaling pathway, ECM-receptor interaction, focal adhesion, metabolic pathways, serotonergic synapse, and tyrosine metabolism might involve in the OSCC progression. The PPI network contains 260 nodes and 655 edges. Then, two important clusters were picked out, and these two cluster are mainly enriched in cell cycle, DNA replication, cellular senescence, IL-17 signaling pathway, and influenza A. 10 genes were identified as hub genes conforming to the degree of connectivity. We then validated the levels of hub genes in ONCOMINE, TCGA and The Human Protein Atlas database. Six genes (SPP1, FN1, CXCL8, BIRC5, PLAUR, and AURKA) were markedly elevated in OSCC and related with poor prognosis. Additionally, genetic analysis demonstrated that hub genes were changed in about 44% OSCC patients and these genetic alternations include amplification, missense mutation and so on.

Importantly, we also focused on finding the therapeutic targets in OSCC. As shown in Fig. 5c, only AURKA, BIRC5, and PLAUR were served as the chemotherapy targets for cancer treatment currently. Therefore, more investigations and clinical trials are required to explore whether the other seven genes (FN1, CXCL8, CXCL10, SPP1, FOXM1, ISG15, and TPX2) could serve as novel therapeutic targets for OSCC patients. According to the results in TIMER website, FOXM1 and TPX2 were regarded as the potential immunotherapeutic targets with future clinical significance. Thus, our analysis may provide valuable clues for the targeted therapy in OSCC.

From construction of the risk gene signature, we found that the nine-gene based risk model could effectively discriminate the OSCC patients with different outcome(P=1.059e-09), and it presents a good performance in prognosis judgement. In addition, the Cox regression analysis further demonstrated that the risk score based on the gene signature is an independent prognostic indicator with the highest HR value than other factors.

Driven-genes play a crucial role in cancer progression through complex pathways and networks. Similar to the findings of our study, it has been reported that secreted phosphoprotein 1 (SPP1) is a cancer-related gene, which presents clearly upregulated level in many cancers [42–44]. Additionally, SPP1 also plays a significant role in extracellular matrix binding [45]. Consistently, SPP1 was enriched in ECM receptor interaction according to KEGG pathway analysis, which plays crucial role in cancer metastasis [46]. Huang et al found that overexpressed SPP1 was linked to carcinogenesis and progression of OSCC [44]. Besides, Fibronectin 1(FN1), predominantly overexpressed in many tumor tissues [47, 48], was also involved in this pathway. Cai et al confirmed that downregulated FN1 could inhibit colorectal carcinogenesis through suppressing proliferation, migration, and invasion [49]. Therefore, SPP1 and FN1 might be potential therapeutic targets for inhibiting OSCC metastasis.

Chemokine (C-X-C motif) ligand 8 (CXCL8) and CXCL10 belong to the chemokine family [50]. Since CXCL8 and CXCL10 integrates with multiple intracellular signaling pathways associated with pro-inflammatory and pro-oncogenic processes, upregulated CXCL8 and CXCL10 are related to carcinogenesis and might predict prognosis of patients [51, 52]. For example, the level of CXCL8 was upregulated in endothelial cells co-cultured with HNSCC, showing that CXCL8 might play a pro-oncogenic role in the pathobiology of tumor cells [53]. Moreover, CXCL8 and CXCL10 are two important modulators in immune response, and they might provide new opportunities for improving immune therapies and enhancing the effectiveness of existing chemotherapies [52, 54].

Plasminogen activator urokinase receptor (PLAUR) is one of glycosyl-phosphatidylinositol (GPI)-anchored membrane proteins [55]. Downregulation of PLAUR could inhibit cancer proliferation and metastasis in several cancers [56]. Consistently, our results showed that PLAUR was considered as a cancer therapeutic target and high expression of PLAUR can predicted poor OS in OSCC patients. Forkhead box protein M1 (FOXM1) is widely participated in the carcinogenesis of several malignances [57]. For example, FOXM1-induced epigenetic signature may serve as ideal biomarkers for early cancer screening in head and neck carcinoma [58]. FOXM1 may also act as a therapeutic target against head and neck carcinoma [59, 60]. Recent study showed that upregulated basal FOXM1 activity predisposes HPV positive HNSCC to WEE1i-induced toxicity [61]. Consistently, we found that FOXM1 is differentially expressed in OSCC patient with different tumor stages, genders, races, and molecular subtypes. Moreover, we were surprised to find that FOXM1 may act as an immune-related therapeutic targets according to the TIMER website. FOXM1 was markedly correlated with PDCD1 (Fig 8b). However, little research has been conducted in this field. Previous study showed that FOXM1 could maintain the dynamic balance between cell apoptosis and proliferation through regulating the essential genes [62]. Thus, FOXM1 may also participate in the PDCD1-regulated cell death. Moreover, the association of FOXM1 with immune cell infiltration may enhance our understanding of the correlationship between FOXM1 and PDCD1[63]. The functions of regulatory T cells (Tregs) in immune regulation is well known, and much is being made of their potential for PDCD1 based therapy [64]. Recently, researchers found that the expression of FOXM1 was positive correlated with FOXP3 (the specific molecular marker of Tregs) and FOXM1 may induce immune suppression via recruiting FOXP3 positive Tregs in cancer therapy [65]. These results highlight the therapeutic potential of FOXM1 in OSCC. Previously, Li et al also found that there was a direct link between PLAUR and FOXM1 [66]. Their results showed that FOXM1 can regulate the level of PLAUR via binding to its promoter. Moreover, the FOXM1-PLAUR axis contributed to colon carcinomas [66]. We therefore supposed that FOXM1-PLAUR signaling might also be promising for designing novel therapeutic drugs for OSCC.

Targeting protein for xenopus kinesin-like protein 2 (TPX2) is involved in the mitotic spindle assembly and cell-cycle progression [67]. Recent studies reported that TPX2 dysregulation was related to the progression of esophageal cancer [68], hepatocellular carcinoma [69], and colorectal cancer [70]. Consistently, increased expression of TPX2 was also observed in OSCC tissues in this study. Moreover, TPX2 is differentially expressed in patients with different tumor stages, genders, races, and molecular subtypes according to UALCAN website. We also analyzed role of TPX2 in tumor immunity and found that TPX2 is involved in the infiltration of B cells, CD4+ T cells, CD8+ T cells, Macrophage cells and Neutrophils cells in OSCC. Importantly, TPX2 has a significant co-expression relationship with CD274, PDCD1LG2, and TOX. Since TOX is a significant regulator for T cell differentiation [71], we believe that TPX2 may participate in OSCC development by regulating TOX molecules.

There were still some limitations in our study. Firstly, our study mainly focused on the biological function of 10 hub genes and did not deeply explore the other DEGs. Therefore, more investigations are required in future. Secondly, because we just utilized ONCOMINE database, TCGA database, and The Human Protein Atlas database to verify the expression of hub genes, the experimental assays are also needed to demonstrate the above results. Finally, the prognostic model should be further validated in large clinical cohort.

## Conclusion

Through conducting in silico analyses, 261 overlapping DEGs were identified in OSCC, which were mainly associated with PI3K-Akt signaling pathway, ECM-receptor interaction, focal adhesion, metabolic pathways, serotonergic synapse, and tyrosine metabolism. We identified 10 hub genes (FN1, CXCL8, CXCL10, SPP1, FOXM1, AURKA, ISG15, PLAUR, TPX2, and BIRC5), which can act as available targets for OSCC treatment. Additionally, we constructed a nine-gene prediction model that can be utilized as the prognostic tool in OSCC. Our study is helpful to explore the carcinogenesis of OSCC. Moreover, these findings can improve the prognosis judgement and targeted therapy of OSCC.

## Supplementary Information

**Additional file 1: Figure S1.** GO and KEGG pathway terms of DEGs in OSCC. **Figure S2.** PPI network of 10 hub genes. **Figure S3.** Validation of hub genes in ONCOMINE database.

**Additional file 2: Table S1.** The enriched pathways in Cluster 1. **Table S2.** The enriched pathways in Cluster 2. **Table S3.** Univariate Cox regression analysis of OS in OSCC patients. **Table S4.** Multivariate Cox regression analysis of OS in OSCC patients.

## Data Availability

All analyzed data related to this paper are included in this paper.

## Abbreviations

DEGs: Differentially expressed genes
logFC: Log‑fold change
STRING: Search tool for the retrieval of interacting genes
OSCC: Oral squamous cell carcinoma
HNSCC: Head and neck squamous cell carcinoma
FOXM1: Forkhead box protein M1
TPX2: Targeting protein for xenopus kinesin-like protein 2
GEO: Gene Expression Omnibus
OS: Overall survival
TCGA: The Cancer Genome Atlas
KEGG: Kyoto Encyclopedia of Genes and Genomes
PPI: Protein-protein interaction
ROC: Receiver operating characteristic
AUC: The area under the ROC curve
