# Supplementary material for: Prognostic biomarkers and therapeutic targets in oral squamous cell carcinoma: a study based on cross-database analysis
Source: Hereditas. 2021 Apr 23;158:15. doi: 10.1186/s41065-021-00181-1 (PMC8066950; doi:10.1186/s41065-021-00181-1)
Supplement: Supplementary file 1 — Additional file 1: Figure S1. GO and KEGG pathway terms of DEGs in OSCC. Figure S2. PPI network of 10 hub genes. Figure S3. Validation of hub genes in ONCOMINE database. [file 41065_2021_181_MOESM1_ESM.docx]

**
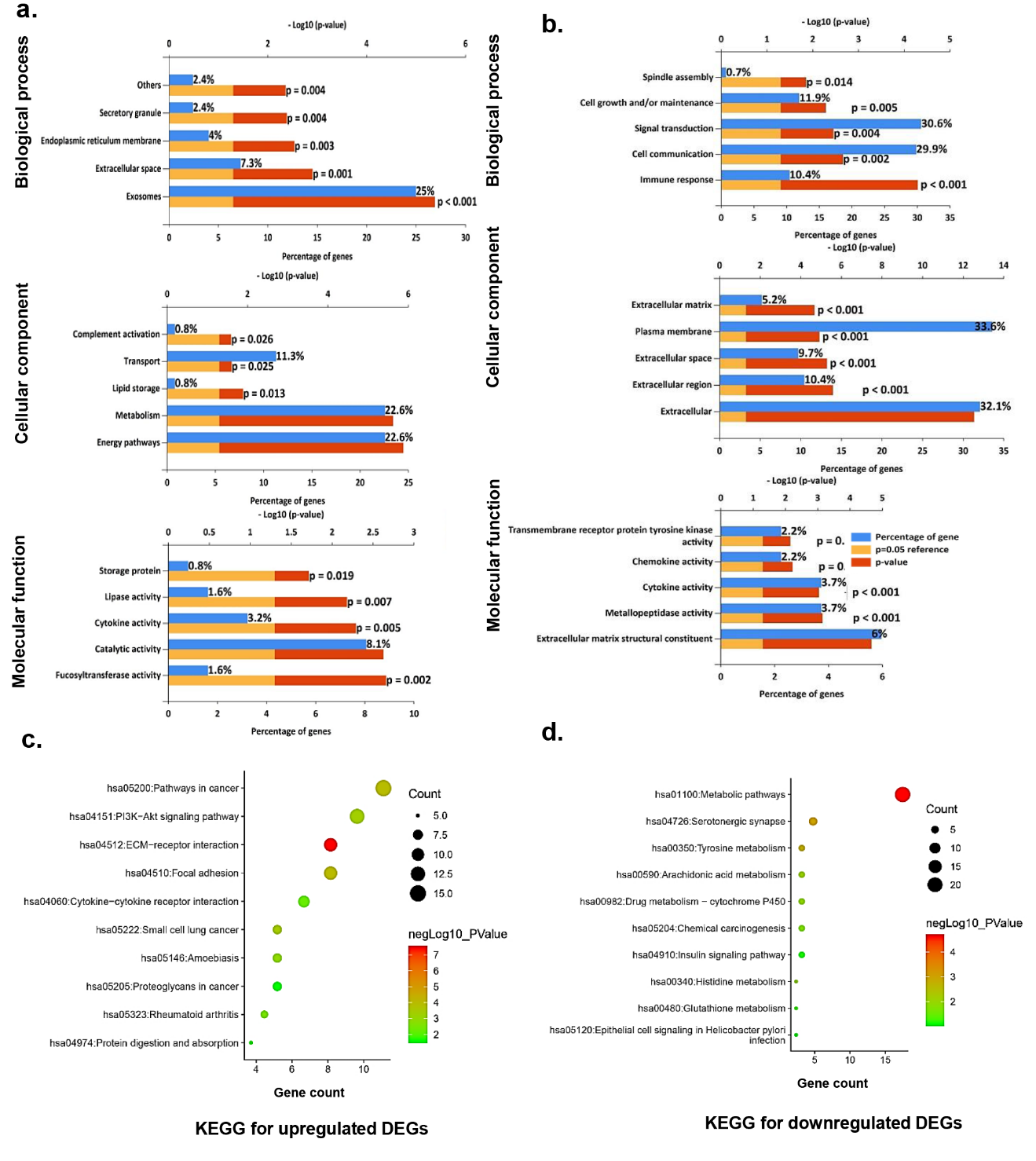
**

**Fig. S1 GO and KEGG pathway terms of DEGs in OSCC.**

a: Top 5 GO terms of upregulated DEGs; b: Top 5 GO terms of downregulated DEGs; c: KEGG pathway for upregulated DEGs; d: KEGG pathway for downregulated DEGs. OSCC, Oral squamous cell carcinoma; DEGs, differentially expressed genes; GO, gene ontology; KEGG, Kyoto encyclopedia of genes and genomes.

**
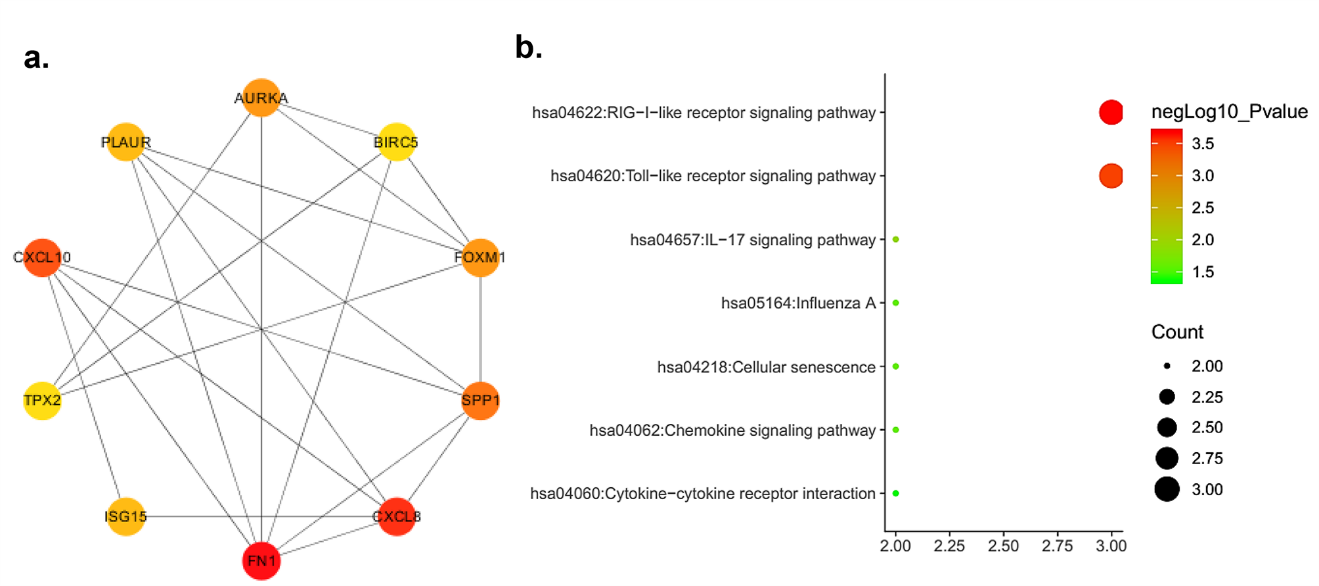
**

**Fig. S2 PPI network of 10 hub genes.**

a: 10 genes (FN1, CXCL8, CXCL10, SPP1, FOXM1, AURKA, ISG15, PLAUR, TPX2, and BIRC5) with higher degree of connectivity were screened as hub genes using Cytoscape (v3.6.1) plugin cytoHubba; b: KEGG pathway of the 10 hub genes. DEGs, differentially expressed genes; PPI, protein-protein interaction; KEGG, Kyoto encyclopedia of genes and genomes.

**
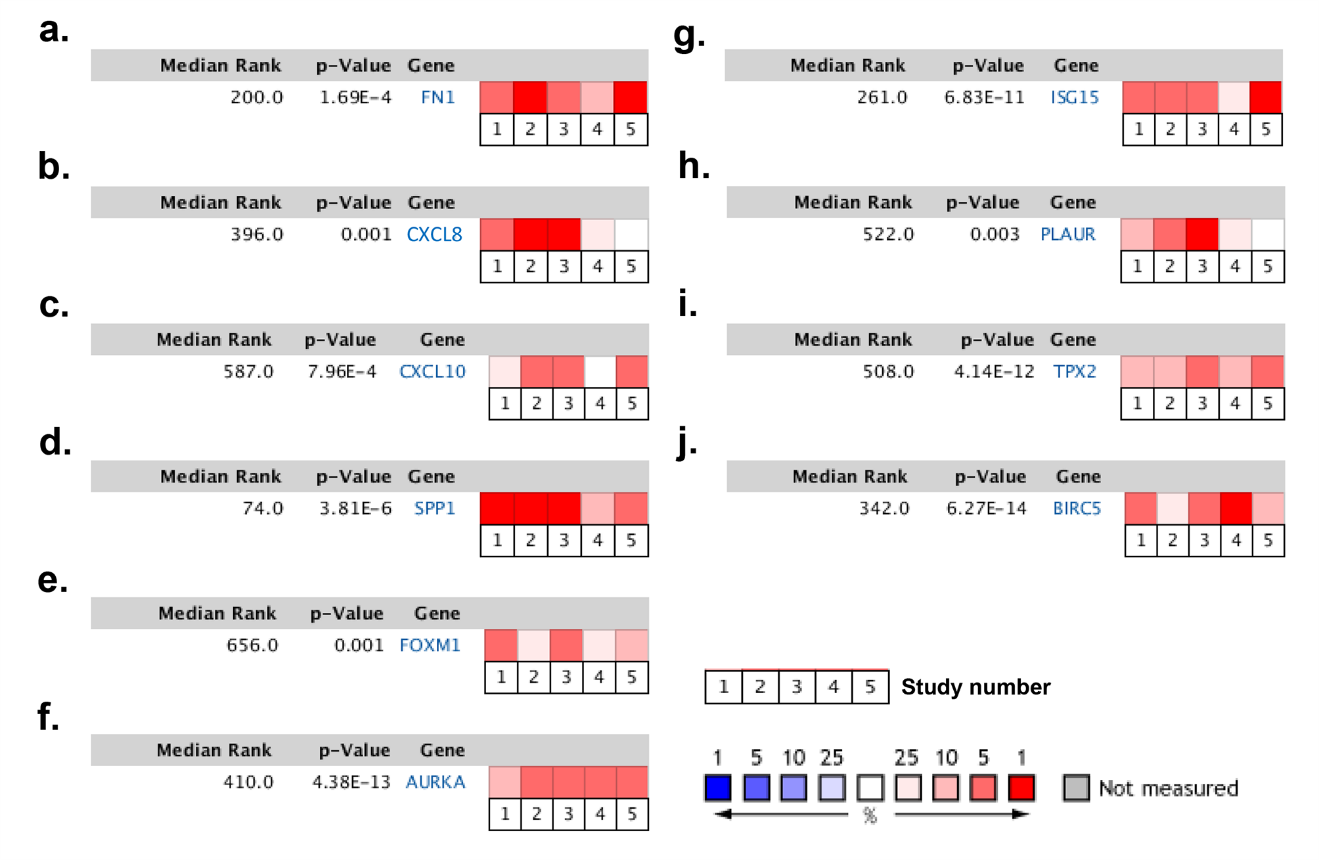
**

**Fig. S3 Validation of hub genes in ONCOMINE database.**

Five ONCOMINE datasets were used to performed the meta‑analysis to compare the mRNA levels of FN1 (a), CXCL8 (b), CXCL10 (c), SPP1 (d), FOXM1 (e), AURKA (f), ISG15(g), PLAUR (h), TPX2 (i), and BIRC5 (j) between OSCC and non-tumor oral tissues. The median rank of 10 hub genes presents as the colored squares (OSCC vs. normal tissue) across the five datasets. Study1 (Cromer A, et al. Oncogene, 2004): HNSCC/OSCC (N=34) vs. Normal Uvula Tissues (N=4); Study2 (Ginos MA, et al. Cancer Res, 2004): HNSCC/OSCC(N=41) vs. Normal Buccal Mucosa Tissues (N=13); Study3 (Peng CH, et al. PLoS One, 2011): OSCC (N=57) vs. Normal Oral Cavity Tissues (N=22); Study4 (Pyeon D, et al. Cancer Res, 2007): OSCC(N=4) vs. Normal Oral Cavity Tissues (N=9); Study5 (Toruner GA, et al. Cancer Genet Cytogenet, 2004): OSCC (N=16) vs. Normal Squamous Cell Tissues (N=4). P<0.05 was set as the significance level for the median rank analysis. OSCC, Oral squamous cell carcinoma; HNSCC, Head and neck squamous cell carcinoma.
