# Supplementary material for: Prognostic biomarkers and therapeutic targets in oral squamous cell carcinoma: a study based on cross-database analysis
Source: Hereditas. 2021 Apr 23;158:15. doi: 10.1186/s41065-021-00181-1 (PMC8066950; doi:10.1186/s41065-021-00181-1)
Supplement: Supplementary file 2 — Additional file 2: Table S1. The enriched pathways in Cluster 1. Table S2. The enriched pathways in Cluster 2. Table S3. Univariate Cox regression analysis of OS in OSCC patients. Table S4. Multivariate Cox regression analysis of OS in OSCC patients. [file 41065_2021_181_MOESM2_ESM.docx]

**Table S1. The enriched pathways in Cluster 1**

| **Term ID** | **Term description** | **Gene count** | **FDR** | **Genes** |
| --- | --- | --- | --- | --- |
| hsa04110 | Cell cycle | 4 | 3.76E-05 | CDC6,CHEK1,MCM2,MCM4 |
| hsa03030 | DNA replication | 2 | 0.0028 | MCM2,MCM4 |
| hsa04218 | Cellular senescence | 2 | 0.0312 | CHEK1,FOXM1 |

**Table S2. The enriched pathways in Cluster 2**

| **Term ID** | **Term description** | **Gene count** | **FDR** | **Genes** |
| --- | --- | --- | --- | --- |
| hsa04657 | IL-17 signaling pathway | 4 | 0.00015 | CXCL10,CXCL8,MMP1,MMP3 |
| hsa05164 | Influenza A | 4 | 0.00074 | CXCL10,CXCL8,OAS2,RSAD2 |
| hsa04610 | Complement and coagulation cascades | 3 | 0.0011 | PLAU,PLAUR,SERPINE1 |
| hsa04622 | RIG-I-like receptor signaling pathway | 3 | 0.0011 | CXCL10,CXCL8,ISG15 |
| hsa05323 | Rheumatoid arthritis | 3 | 0.0011 | CXCL8,MMP1,MMP3 |

**Table S3 Univariate Cox regression analysis of OS in OSCC patients**

| Genes | HR | p-value |
| --- | --- | --- |
| SLCO1B3 | 1.198084(1.028651-1.395425) | 0.020175 |
| TENM2 | 1.132033(1.017666-1.259251) | 0.022475 |
| TEX101 | 0.58283(0.403243-0.842396) | 0.004072 |
| MFAP4 | 0.892839(0.808187-0.986357) | 0.025731 |
| DSG2 | 1.188206(1.042404-1.354401) | 0.009831 |
| HMGA2 | 1.156437(1.026684-1.302587) | 0.016681 |
| CTSL | 1.174747(1.015828-1.358527) | 0.029876 |
| GATM | 0.820336(0.699546-0.961982) | 0.014814 |
| PLAU | 1.213479(1.069604-1.376706) | 0.002656 |
| FOXD1 | 1.264246(1.065367-1.500251) | 0.007252 |
| SCG5 | 1.215924(1.039608-1.422143) | 0.014448 |
| FXYD5 | 1.180952(1.025695-1.35971) | 0.020737 |
| ITGA5 | 1.207443(1.061094-1.373975) | 0.004243 |
| LRRK2 | 0.641349(0.432093-0.951943) | 0.027498 |
| KLB | 0.100248(0.016931-0.593568) | 0.011252 |
| AURKA | 1.247717(1.021363-1.524236) | 0.03024 |
| ADA | 1.320589(1.10653-1.576057) | 0.002057 |
| HLF | 0.791095(0.668558-0.93609) | 0.006351 |
| BOC | 0.671046(0.524723-0.858173 | 0.001479 |
| CXCL13 | 0.88884(0.814238-0.970276) | 0.008424 |
| SCNN1B | 0.886915(0.791438-0.99391) | 0.038915 |
| SERPINE1 | 1.125864(1.029865-1.230811) | 0.009131 |
| DCBLD1 | 1.20813(1.027362-1.420705) | 0.022231 |
| SCARA5 | 0.813638(0.663245-0.998132) | 0.047941 |
| TNFRSF12A | 1.216957(1.053663-1.405556) | 0.007561 |
| FAM3D | 0.895346(0.813368-0.985586) | 0.024051 |
| PAQR8 | 0.740424(0.582109-0.941794) | 0.014343 |
| ITGA3 | 1.165175(1.021224-1.329418) | 0.02308 |
| AR | 0.62374(0.404132-0.962683) | 0.03303 |
| INHBA | 1.122078(1.021644-1.232385) | 0.016061 |
| FST | 1.157173(1.047575-1.278238) | 0.004034 |
| ZNF114 | 1.226847(1.040643-1.44637） | 0.01492 |
| PBX1 | 0.819613(0.689994-0.973582) | 0.023526 |
| FAM189A2 | 0.778842(0.61596-0.984796) | 0.036803 |
| SOCS1 | 0.827956(0.713097-0.961315) | 0.013221 |
| RALGPS1 | 0.699243(0.501533-0.974894) | 0.034865 |
| MIR99AHG | 0.424275(0.249389-0.721802) | 0.001565 |
| FUT6 | 0.860447(0.741585-0.998359) | 0.047523 |
| CYP27B1 | 1.173156(1.010454-1.362056) | 0.036038 |
| GAS7 | 0.808739(0.671945-0.973381) | 0.024747 |
| CYP4F12 | 0.784143(0.633454-0.97068) | 0.025531 |
| STC2 | 1.303003(1.140846-1.48821) | 9.49E-05 |

**Table S4 Multivariate Cox regression analysis of OS in OSCC patients**

| **Gene** | **coef** | **HR** | **p-value** |
| --- | --- | --- | --- |
| TEX101 | -0.32654 | 0.721413(0.499646-1.041612) | 0.081435 |
| DSG2 | 0.102566 | 1.108011(0.965362-1.271739) | 0.144667 |
| SCG5 | 0.150217 | 1.162086(0.980354-1.377506) | 0.0834 |
| ADA | 0.186215 | 1.204681(0.99786-1.45437) | 0.052664 |
| BOC | -0.29365 | 0.745535(0.567854-0.978813) | 0.034504 |
| SCARA5 | -0.26023 | 0.770875(0.610384-0.973564 | 0.028895 |
| FST | 0.107955 | 1.113998(1.006239-1.233296) | 0.037544 |
| SOCS1 | -0.26676 | 0.76586(0.649373-0.903242) | 0.00153 |
| STC2 | 0.15679 | 1.169749(1.016972-1.345478) | 0.028117 |
